# Intestinal parasite infections and associated risk factors in communities exposed to wastewater in urban and peri-urban transition zones in Hanoi, Vietnam

**DOI:** 10.1186/s13071-016-1809-6

**Published:** 2016-10-10

**Authors:** Samuel Fuhrimann, Mirko S. Winkler, Phuc Pham-Duc, Dung Do-Trung, Christian Schindler, Jürg Utzinger, Guéladio Cissé

**Affiliations:** 1 Swiss Tropical and Public Health Institute, Basel, Switzerland; 2 University of Basel, Basel, Switzerland; 3 Center for Public Health and Ecosystem Research, Hanoi School of Public Health, Hanoi, Vietnam; 4 Department of Parasitology, National Institute of Malaria, Parasitology, and Entomology, Hanoi, Vietnam

**Keywords:** Helminth, Intestinal protozoa, Peri-urban farming, Urban farming, Vietnam, Wastewater

## Abstract

**Background:**

Infections with intestinal parasites (helminths and intestinal protozoa) are endemic in Southeast Asia and inappropriate management and reuse of wastewater might exacerbate the risk of human infections. In rapidly growing urban settings, little is known about the extent of intestinal parasite infections. We assessed the point-prevalence and risk factors of intestinal parasite infections in population groups differently exposed to wastewater in urban and peri-urban transition zones in Hanoi, the capital of Vietnam.

**Methods:**

A cross-sectional survey was carried out between April and June 2014 in people aged ≥ 18 years at risk of wastewater exposure from To Lich River: workers maintaining wastewater treatment facilities; urban farmers reusing wastewater; and urban dwellers at risk of flooding events. For comparison, two peri-urban population groups living in close proximity to the Red River were chosen: farmers using river water for irrigation purposes; and people living in the same communities. A single stool sample was subjected to Kato-Katz and formalin-ether concentration methods for the diagnosis of helminth and intestinal protozoa infections. A questionnaire was administered to determine risk factors and self-reported signs and symptoms.

**Results:**

A total of 681 individuals had complete data records. Highest point-prevalence rates of intestinal parasite infections were observed for peri-urban farmers (30 %). Hookworm and *Trichuris trichiura* were the predominant helminth species (25 % and 5 %, respectively). Peri-urban farmers were at higher odds of infection with intestinal parasites than any other groups (adjusted odds ratio 5.8, 95 % confidence interval 2.5 to 13.7). Lack of access to improved sanitation and not receiving deworming within the past 12 months were associated with higher infection risk, while higher educational attainment and socioeconomic status were negatively associated with intestinal parasite infections.

**Conclusions:**

Our results suggest that exposure to wastewater was not directly associated with infection with helminths and intestinal protozoa in different population groups in Hanoi. These findings might be explained by a high level of awareness of health risks and access to safe sanitary infrastructure in urban areas. The high prevalence rates observed in peri-urban farmers call for specific interventions targeting this population group.

**Electronic supplementary material:**

The online version of this article (doi:10.1186/s13071-016-1809-6) contains supplementary material, which is available to authorized users.

## Background

In Southeast Asia, infections with intestinal parasites (e.g. helminths and intestinal protozoa) cause a considerable public health burden [1, 2]. Despite efforts to control morbidity and interrupt transmission, infection with soil-transmitted helminths (*Ascaris lumbricoides*, hookworm, *Strongyloides stercoralis* and *Trichuris trichiura*) are common and show geographic, demographic, socioeconomic and cultural differences within and across countries of Cambodia, Lao People’s Democratic Republic (PDR) and Vietnam [3–5]. In urban areas, socioeconomic development, including improvements in sanitation and water infrastructures are thought to be associated with a decline in the prevalence and intensity of intestinal parasites over the past decades [6–8]. However, in rural areas and deprived urban and peri-urban settings, access to clean water and improved sanitation remains insufficient and is an important risk factor for infections with helminth and intestinal protozoa [9, 10]. Additionally, reuse of wastewater and faeces in agriculture and aquaculture might contribute to the transmission of intestinal parasites [2, 11].

Hanoi, the capital of Vietnam, has undergone considerable economic growth since the end of the Vietnam War in 1975, resulting in a change in lifestyles and increased living standards. Moreover, population growth and rural-urban migration led to an expansion of the city boundaries [12]. Due to rapid urbanization, improved access to health care and awareness campaigns are available (i.e. yearly deworming of school-aged children and hygiene campaigns such as “eating cooked food and drinking boiled water”), which decreased prevalence of intestinal parasitic infections [13]. However, increasing volumes of domestic waste, mixed with chemical and microbial pollutants, have increased the heterogeneity in exposure to such pollutants and pathogens [14, 15]. Especially for urban and peri-urban transition zones around Hanoi, it is crucial to ensure access to basic water and sanitation infrastructures. Moreover, guidance on safe management and reuse of wastewater is needed [6, 7, 16]. It is conceivable that increasing volumes of wastewater might exacerbate the spread of intestinal parasites, enteric bacteria and viruses [16, 17]. Moreover, past extreme weather events, such as heavy rains, jeopardized the proper functionality of Hanoi’s sanitation systems, with likely adverse health outcomes [18].

In urban and peri-urban areas of Hanoi, an estimated 650,000 farmers reuse wastewater in agriculture and aquaculture to supply the 6.7 million people living in the city with fresh vegetables and fish [19]. Use of wastewater comes at low cost for water and nutrients, and hence provides an important livelihood opportunity for farming communities [20]. Of note, lack of sanitation facilities and use of human excreta in such communities were shown to be a major risk factor for intestinal parasite infections. Moreover, diarrhoeal and skin diseases have been associated to occupational contact with wastewater [13, 21–24]. In more rural communities, the occupational exposure to Hanoi’s reused wastewater has also been associated with *A. lumbricoides* and *T. trichiura* infections [2]. Thus, it is commonly observed in urban communities that the prevalence rates of intestinal parasitic infections are lower than in peri-urban and rural areas [1]. Over the past decade, a number of studies indicated levels of microbial and chemical pollution above national and international safety standards in the environment [15, 25–28]. Thus, pollution reduction may not be sufficient to allow for safe reuse of wastewater for agriculture and aquaculture [29].

As the city of Hanoi expanded rapidly, with annual population growth rates of up to 3.5 %, timely data on prevalence and risk factors of infection with helminths and intestinal protozoa are needed to understand the effect of urbanization in urban and peri-urban transition zones [12]. Surveys investigating prevalence rates and risk factors for parasitic diseases, diarrhoea, skin and eye infections in the urban and peri-urban environment around Hanoi are dating back to 2005 [13, 21–24]. Such data will help to effectively plan public health interventions and justify investments in sanitary infrastructures [16, 30]. The objective of the present study was to assess the prevalence rates and risks factors for intestinal parasite infections in different population groups exposed to wastewater reuse activities in Hanoi.

## Methods

### Study design and participants

**Fig. 1:**
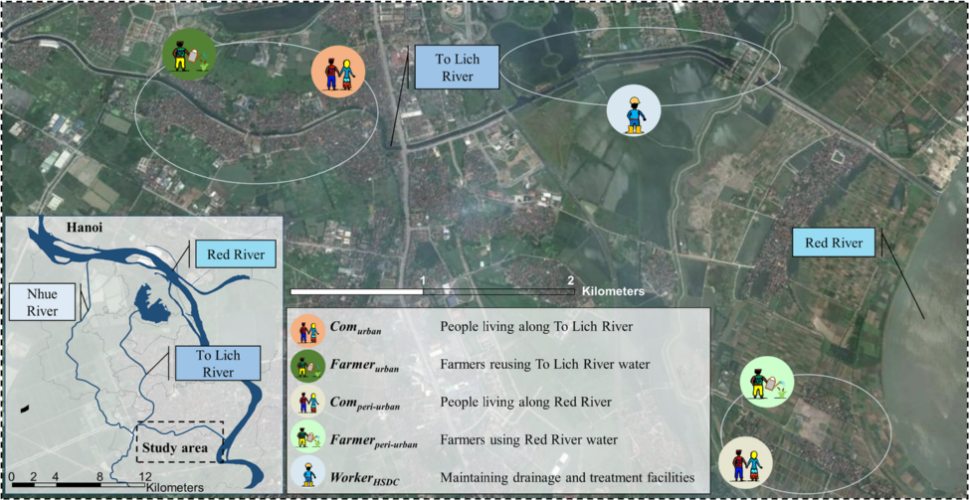
Map of Hanoi showing the study area and the five exposure groups in the Than Tri district. (Map data ©2015 Google)

A cross-sectional survey was conducted between April and June 2014. The study was undertaken in the southern part of Hanoi, along To Lich River (main open storm water and drainage channel of the city) and Red River (natural river stemming from the People's Republic of China that is discharged in the Gulf of Tonkin). These rivers receive most of the city’s wastewater, managed by Hanoi Sewerage and Drainage Company (HSDC). However, water quality differs considerably: while water of the To Lich River is not allowing for the safe reuse of wastewater in agriculture and aquaculture according the World Health Organization (WHO) guidelines, the Red River water quality is within tolerable limits colony forming unit (CFU) total coliforms and *Escherichia coli* (4.2 × 10^6^ CFU/100 ml and 1.7 × 10^4^ CFU/100 ml, respectively). Helminth eggs were only found in To Lich River (0.1 egg/l), which however is still within the WHO tolerable concentration for safe reuse [16, 29]. Particular emphasis was placed to the wastewater reuse in agriculture and aquaculture in urban and peri-urban transition zones of the districts Hoang Mai and Thanh Tri (geographical coordinates: 21°01'42.5"N, 105°51'15.0"E) (Fig. 1). A detailed description of the study system and water quality of the rivers is published elsewhere [29].

The study enrolled adults (aged ≥ 18 years) living and working in urban or peri-urban areas in the two districts. According to the level of exposure to wastewater, the study participants were stratified into five population groups: three exposed to wastewater from To Lich River (i-iii); and two comparison groups living along Red River without direct exposure to urban wastewater (iv and v):

(i) “*Com*_*urban*_”, people living in the urban to peri-urban transition zone of Hanoi, in Bang B village or Tam Hiep commune along To Lich River who are potentially exposed to wastewater while flooding events occur during the rainy season. The communities are located in Hoang Mai and Thanh Tri district, respectively (geographical coordinates: 20°57'17.54"N, 105°49'42.48"E), and are prone to rapid demographic transition, industrial development and land use change.
(ii) “*Farmer*_*urban*_”, urban farmers living in Bang B village or Tam Hiep commune reusing wastewater from To Lich River. A large part of the community members (33 %) are involved in agriculture (e.g. rice, morning glory, neptunia and watercress mainly) or aquaculture activities [31].
(iii) “*Worker*_*HSDC*_”, workers from HSDC maintaining drainage channels and operating the Yen So treatment plants along To Lich River.
(iv) “*Com*_*peri-urban*_”, people living in Duyen Ha commune (comparison group). The commune represents a typical peri-urban community along Red River with poor sanitation and drinking water systems. The commune belongs to Thanh Tri district and is located approximately 5 km from the outskirt of Hanoi (geographical coordinates: 20°55'42.37"N, 105°52'23.32"E).
(v) “*Farmer*_*peri-urban*_”, farmers living in Duyen Ha commune using the irrigation water from Red River (comparison group). About 38 % of the people work in agriculture.

Sample size was calculated by aiming at a power of 95 %, to ensure that a reduction in effective exposure variance by 35 % following confounder adjustment would still leave 80 % power. Our assumptions were that the prevalence of intestinal parasite infections is at least 20 % in *Com*_*peri-urban*_ and the odds ratio (OR) of *Farmer*_*urban*_, and *Worker*_*HSDC*_ to *Com*_*peri-urban*_ is at least 2.5. We also assumed that the final sample size might be reduced by 15 % due to loss to follow-up. Hence, our intended sample size was 1,025 (*Com*_*urban*_, *n* = 250; *Farmer*_*urban*_, *n* = 250; *Com*_*peri-urban*_, *n* = 175; *Farmer*_*peri-urban*_, *n* = 175; and *Worker*_*HSDC*_, *n* = 150).

The following inclusion and exclusion criteria were applied. First, households were randomly selected from two separate lists (one for farming and one for all non-farming households in the community) readily available from the communal people committees. All listed households were numbered and the appropriate number selected using a random number list from Excel. All individuals in the selected households were invited to participate in the survey. If they were willing to participate, one person per household (household heads or adults living permanently in the household) was selected for a questionnaire interview at a convenient time at the community health station. Participants were provided with a stool container and asked to return a filled container the day of the interview with her or his own morning stool sample. To select members of *Worker*_*HSDC*_, the HSDC headquarter mobilized and informed the workers and randomly selected them from the existing staff list. *Worker*_*HSDC*_ were then invited to come on a fixed day for the interview along with a fresh morning stool sample to the health station of the HSDC the day after the interview.

### Procedures

We employed a questionnaire to determine exposure pathways to wastewater, potential confounding factors (e.g. demographic and socioeconomic), risk variables (e.g. water, sanitation, hygiene and occupation) and self-reported signs and symptoms. Our questionnaire had previously been validated in a study in Uganda [32]. The questionnaire was translated into Vietnamese, and further adapted to the Hanoi context and pre-tested among five farmers and five community members not otherwise involved in the current study. Research assistants entered data directly into tablet computers (Samsung Galaxy note 10.1 N8010) via a data entry mask using Open Data Kit (http://opendatakit.org).

Participants were invited to provide a fresh morning stool that was subjected to the Kato-Katz technique (duplicate thick smears, using standard 41.7 mg template) [33] and a formalin-ether concentration technique (FECT) [34] for the diagnosis of helminths (*A. lumbricoides*, hookworm, *T. trichiura* and other helminths) and intestinal protozoa (*Blastocystis hominis*, *Chilomastix mesnili*, *Endolimax nana*, *Entamoeba coli*, *Entamoeba histolytica*/*E. dispar*, *Entamoeba hartmanni*, *Giardia intestinalis* and *Iodamoeba bütschlii*). Kato-Katz thick smear and FECT readings were double-entered and cross-checked.

### Statistical analysis

Helminth- and intestinal protozo-specific proportions were compared between the five exposure groups, using Pearson’s *χ* ^2^ test. Univariate logistic regression was applied to investigate for potential associations between nine dependent variables, i.e. infections with (i) any intestinal parasite; (ii) soil-transmitted helminth; (iii) intestinal protozo; (iv) *A. lumbricoides*; (v) hookworm; (vi) *T. trichiura*; (vii) 14-day diarrhoea prevalence; (viii) skin problems; and (ix) eye problems), and 20 independent variables (e.g. exposure groups, sex and age). A measure of socioeconomic status was derived, based on an asset index using principal components analysis (PCA), with participants grouped into four categories, as summarised in Table 1 (most poor, poor, less poor and least poor) [35]. Our multivariate core model included the categorical exposure variables sex, age, educational attainment and socioeconomic status [9, 36]. We then added risk factors that had a *P*-value lower than 0.2 (using likelihood ratio test) in the univariate analyses. Of note, a univariate or multivariate analysis was only conducted if the number of respective cases was above 50 or 70, respectively.

**Table 1:** Demographic and socioeconomic characteristics of the participants enrolled in the cross-sectional survey, stratified by five exposure groups in the Than Tri district, Hanoi, between April and June 2014

Demographic and socioeconomic characteristics/Exposure groups^a^	*Com*_*peri-urban*_		*Com*_*urban*_		*Farmer*_*peri-urban*_		*Farmer*_*urban*_		*Worker*_*HSDC*_	
	*N* = 101		*N* = 170		*N* = 129		*N* = 153		*N* = 128	
	*n*	%	*n*	%	*n*	%	*n*	%	*n*	%
Sex										
Female	85	84.2	134	78.8	105	81.4	132	86.3	58	45.3
Male	16	15.8	36	21.2	24	18.6	21	13.7	70	54.7
Age categories (years) (mean ± SD)	50.0 ± 15.6		45.7 ± 14.5		48.7 ± 11.1		52.6 ± 10.6		41.2 ± 10.7	
Educational attainment										
Never went to school	3	3.0	7	4.1	0	0.0	5	3.3	0	0.0
Primary school	13	12.9	16	9.4	19	14.7	42	27.5	2	1.6
Secondary school	47	46.5	70	41.2	76	58.9	76	49.7	37	28.9
Tertiary school	15	14.9	59	34.7	31	24.0	27	17.6	73	57.0
University degree	23	22.8	18	10.6	3	2.3	3	2.0	16	12.5
Socioeconomic status^b^										
Most poor	28	27.7	31	18.2	51	39.5	33	21.6	12	9.4
Poor	22	21.8	49	28.8	42	32.6	48	31.4	17	13.3
Less poor	17	16.8	41	24.1	25	19.4	34	22.2	56	43.8
Least poor	34	33.7	49	28.8	11	8.5	38	24.8	43	33.6
How many people live in your household (mean ± SD)	4.7 ± 2.0		4.6 ± 1.7		4.3 ± 1.9		5.1 ± 2.6		5.3 ± 8.5	
Living at the same place (years) (mean ± SD)	34.4 ± 21.3		37.7 ± 19.8		37.8 ± 19.5		53.3 ± 56.3		34.0 ± 14.5	

^a^Exposure groups: *Com*_*peri-urban*_: people living in the peri-urban commune Duyen Ha, 5 km away from the city along the Red River; *Com*_*urban*_: people living in the urban area of Hanoi, in Bang B village or Tam Hiep commune along the To Lich River and potential exposed to wastewater; *Farmer*_*peri-urban*_: peri-urban farmers living in Duyen Ha commune using the irrigation water from Red River, wells or local drains, which are not contaminated with the city’s wastewater; *Farmer*_*urban*_: urban farmers living in Bang B village or Tam Hiep commune reusing wastewater from To Lich River; and *Worker*_*HSDC*_: workers from Hanoi Sewerage and Drainage Company (HSDC) maintaining drainage channels and operating the Yen So treatment plants

^b^Derived using principal components analysis (PCA) of the following 11 ownership items: radio, TV, mobile phone, fridge, computer, bicycle, motorbike, car, electricity, running water and latrine

ORs were reported to compare risks. Differences and associations were considered as statistically significant if their *P*-values were below 0.05 and as indicating a trend if *P*-values were between 0.05 and 0.1. Statistical analyses were done using STATA version 12.0 (Stata Corporation; College Station, USA).

## Results

**Fig. 2:**
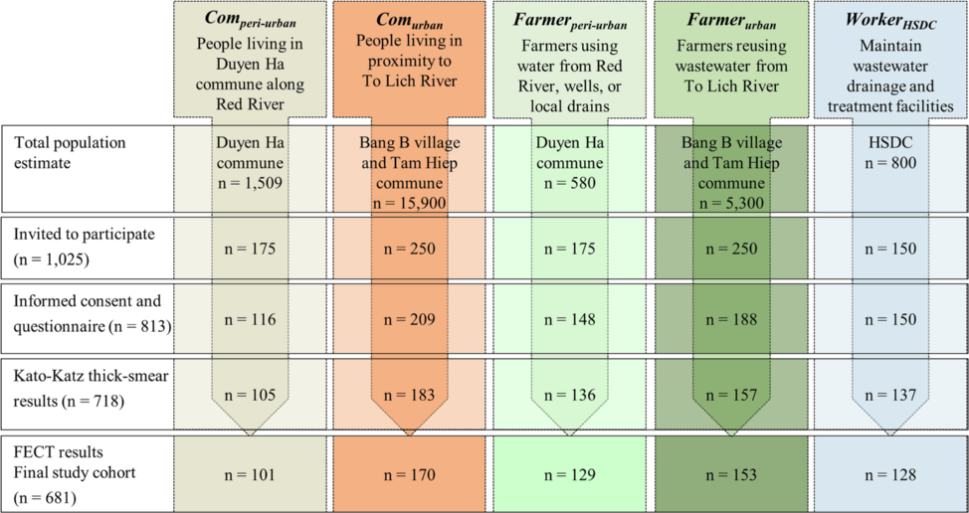
Flow chart indicating the enrolment of study participants and compliance, stratified into exposure groups in the cross-sectional survey in the Than Tri district, Hanoi, between April and June 2014

Among 1,025 people invited, 813 fulfilled our inclusion criteria, provided written informed consent and completed the questionnaire interview (Fig. 2). Stool samples were provided by 718 individuals that were subjected to Kato-Katz thick smear examination. Due to insufficient volumes of stool provided, only 681 of the samples were subjected to FECT. These 681 individuals were considered as the final study cohort, composed of 170 *Com*_*urban*_, 153 *Farmer*_*urban*_, 129, *Farmer*_*peri-urban*_, 128 *Worker*_*HSDC*_ and 101 *Com*_*peri-urban*_.

Table 1 summarises the demographic (sex, age, educational attainment, people per household, living duration at the same place) and socioeconomic characteristics, stratified by the five population groups. In brief, females accounted for 79 % and more in all exposure groups, expect for *Worker*_*HSDC*_ (45 %). Most of the participants (>60 %) were aged above 40 years and attended in minimum secondary school. Socioeconomic status was highest in *Worker*_*HSDC*_ and *Com*_*peri-urban*_ with 34 % classified as “least poor” in both groups. The lowest socioeconomic status was observed in *Farmer*_*peri-urban*_ with 40 % classified as “most poor”. On average, between 4.3 and 5.3 people live in a household. Two-thirds of the participants (65 %) reported that they lived in the study area for at least ten years.

Risk factors for intestinal parasite infections, such as perceived exposure to wastewater, access to sanitation, drinking water and bath water and deworming practise are shown in Table 2. Almost 90 % of the participants exposed to wastewater perceived wastewater as polluted water, which causes ill-health and environmental risks (*Com*_*urban*_, *Farmer*_*urban*_ and *Worker*_*HSDC*_), while 26 to 28 % *Farmer*_*peri-urban*_ perceived no health and environmental risks due to wastewater. Past flooding of the working area was most frequently reported among *Farmer*_*urban*_ (39 %) and *Worker*_*HSDC*_ (41 %). Overall, 96 % of participants reported to have a toilet at home, whereas 15 % of the *Farmer*_*peri-urban*_ had no accesses to sanitation and thus perform open defecation. Self-reported deworming drugs within the past six months ranged between 7 % (*Farmer*_*urban*_) and 13 % (*Com*_*peri-urban*_).

**Table 2:** Water, sanitation and hygiene (WASH) specific risk factors of the participants enrolled in a cross-sectional survey, stratified by the five exposure groups in the Than Tri district, Hanoi, between April and June 2014

Risk factors related to water, sanitation and hygiene/ Exposure groups^a^	*Com*_*peri-urban*_		*Com*_*urban*_		*Farmer*_*peri-urban*_		*Farmer*_*urban*_		*Worker*_*HSDC*_	
	*N* = 101		*N* = 170		*N* = 129		*N* = 153		*N* = 128	
	*n*	%	*n*	%	*n*	%	*n*	%	*n*	%
Wastewater is …										
polluted water	87	86.1	162	95.3	111	86.0	143	93.5	126	98.4
causing health issues	83	82.2	149	87.6	96	74.4	141	92.2	126	98.4
causing environmental issues	83	82.2	149	87.6	93	72.1	135	88.2	127	99.2
Exposure to wastewater (water from rivers or lakes around Hanoi) while …										
flooding of living area	0	0.0	5	2.9	3	2.3	2	1.3	17	13.3
flooding of working area	1	1.0	9	5.3	21	16.3	59	38.6	53	41.4
washing clothes	0	0.0	0	0.0	0	0.0	1	0.7	2	1.6
cleaning of a fish pond	0	0.0	1	0.6	0	0.0	5	3.3	15	11.7
fishing	3	3.0	7	4.1	3	2.3	6	3.9	13	10.2
swimming	1	1.0	3	1.8	2	1.6	0	0.0	6	4.7
Toilet facility at household										
Flush toilet	94	93.1	167	98.2	108	83.7	147	96.1	127	99.2
Pit latrine	6	5.9	6	3.5	2	1.6	6	3.9	0	0.0
No facility (defecation in the open)	1	1.0	1	0.6	19	14.7	1	0.7	1	0.8
Toilet facility at work										
Flush toilet	91	90.1	150	88.2	61	47.3	47	30.7	39	30.5
Pit latrine	6	5.9	14	8.3	4	3.1	4	2.6	68	53.1
No facility (defecation in the open)	4	4.0	6	3.5	64	49.6	102	66.7	21	16.4
Household with tap water	82	81.2	164	96.5	102	79.1	148	96.7	126	98.4
Source of drinking water (multiple answers possible)										
Bottled water	40	39.6	65	38.2	36	27.9	41	26.8	61	47.7
Tap water	60	59.4	149	87.6	73	56.6	136	88.9	113	88.3
Rain water	14	13.9	5	2.9	18	14.0	9	5.9	7	5.5
Bore hole water	31	30.7	7	4.1	43	33.3	4	2.6	1	0.8
Source of bathing water (multiple answers possible)										
Tap water	73	72.3	150	88.2	55	42.6	70	45.8	123	96.1
Rain water	6	5.9	2	1.2	11	8.5	18	11.8	8	6.3
Bore hole water	42	41.6	16	9.4	76	58.9	9	5.9	12	9.4
Well water	2	2.0	0	0.0	4	3.1	0	0.0	2	1.6
Water from lakes or rivers	0	0.0	1	0.6	5	3.9	48	31.4	19	14.8
Preventive chemotherapy received in the past										
< 6 months	13	12.9	15	8.8	15	11.6	11	7.2	15	11.7
6 to < 12 months	9	14.1	24	14,1	12	9.3	12	7.8	20	15.6
> 12 months	75	71.2	121	71.2	96	74.4	114	74.5	87	68.0
Never took deworming	4	4.0	10	5.9	6	5.7	16	10.5	6	4.7

^a^Exposure groups: *Com*_*peri-urban*_: people living in the peri-urban commune Duyen Ha, 5 km away from the city along the Red River; *Com*_*urban*_: people living in the urban area of Hanoi, in Bang B village or Tam Hiep commune along the To Lich River and potential exposed to wastewater; *Farmer*_*peri-urban*_: peri-urban farmers living in Duyen Ha commune using the irrigation water from Red River, wells or local drains, which are not contaminated with the city’s wastewater; *Farmer*_*urban*_: urban farmers living in Bang B village or Tam Hiep commune reusing wastewater from To Lich River; and *Worker*_*HSDC*_: workers from Hanoi Sewerage and Drainage Company (HSDC) maintaining drainage channels and operating the Yen So treatment plants

Table 3 shows occupational conditions (employment status, working hours, etc.) and protective factors (personal protective equipment) for *Farmer*_*peri-urban*_, *Farmer*_*urban*_ and *Worker*_*HSDC*_. While all *Worker*_*HSDC*_ reported to be officially contracted, 90 % and 91 % of the *Farmer*_*peri-urban*_ and *Farmer*_*urban*_ lacked an official employment status, respectively. More than 90 % of all *Worker*_*HSDC*_ used different personal equipment (e.g. gloves, boots, uniform) for self-protection against wastewater exposure, while approximately 80 % farmers owned boots and gloves.

**Table 3:** Risk factors related to the occupation of workers and farmers enrolled in the cross-sectional survey in the Than Tri district, Hanoi, between April and June 2014

Risk factors related to occupation/Exposure groups^a^	*Farmer*_*peri-urban*_		*Farmer*_*urban*_		*Worker*_*HSDC*_	
	*N* = 129		*N* = 153		*N* = 128	
	*n*	%	*n*	%	*n*	%
Employed	13	10.1	13	8.5	128	100
Retired	11	8.5	16	10.5	0	0
Duration worked in the current job (mean ± SD)	30.3 ± 12.9		36.9 ± 13.5		15.3 ± 9.1	
Days worked per week (mean ± SD)	6.5 ± 1.2		5.5 ± 2.1		6.2 ± 0.6	
Hours worked per week (mean ± SD)	39.8 ± 17.3		35.9 ± 23.2		50.0 ± 4.0	
Possession of personal protective equipment						
Gloves	106	82.2	113	73.9	117	91.4
Boots	107	82.9	131	85.6	110	85.9
Uniform/cotton overall	28	21.7	11	7.2	120	93.8
Rain coat with boots	29	22.5	48	31.4	120	93.8
Rain coat without boots	36	27.9	58	37.9	84	65.6
Long sleeves	97	75.2	137	89.5	48	37.5
Helmet	3	2.3	1	0.7	117	91.4
Soft hat (baseball cap)	24	18.6	37	24.2	7	5.5
Vietnamese hat	111	86.0	141	92.2	4	3.1
Face mask	110	85.3	105	68.6	121	94.5
Application of...						
Pesticides	113	87.6	117	76.5	na^b^	
Fertilizer	122	94.6	146	95.4	na	

^a^Exposure groups: *Farmer*_*peri-urban*_: peri-urban farmers living in Duyen Ha commune using the irrigation water from Red River, wells or local drains, which are not contaminated with the city’s wastewater; *Farmer*_*urban*_: urban farmers living in Bang B village or Tam Hiep commune reusing wastewater from To Lich River; and *Worker*_*HSDC*_: workers from Hanoi Sewerage and Drainage Company (HSDC) maintaining drainage channels and operating the Yen So treatment plants

^b^na, not applicable for sanitation workers

**Fig. 3:**
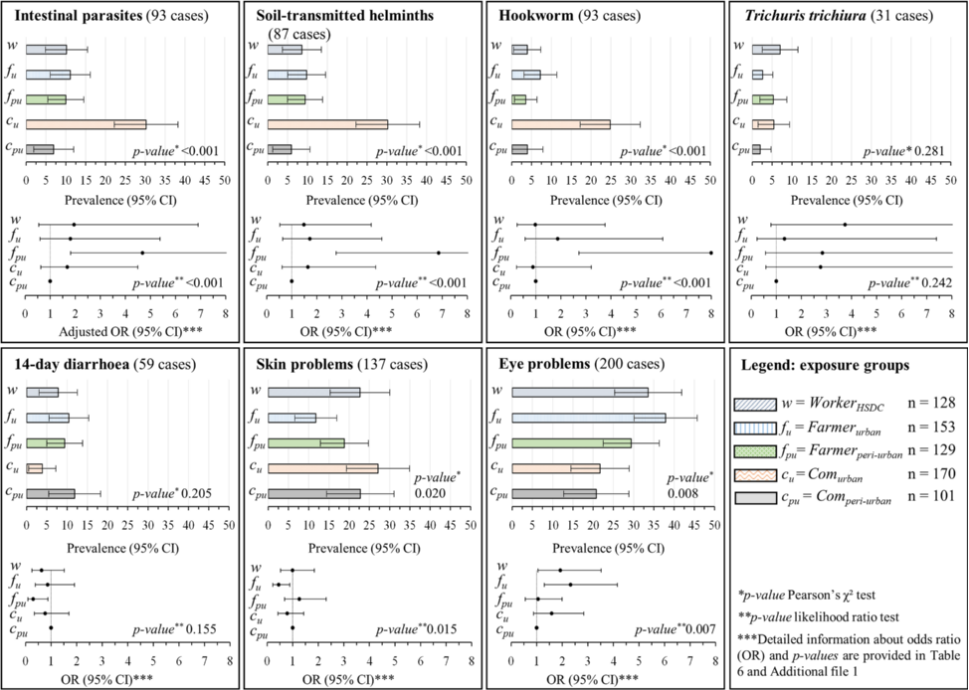
Prevalence rates and adjusted odds ratios (OR) with 95 % confidence intervals (CIs) for infection with any intestinal parasite, soil-transmitted helminth, hookworm, *Trichuris trichiura* and self-reported diarrhoea, skin problems and eye problems in a cross-sectional survey in the Than Tri district, Hanoi, between April and June 2014. Data for (i)“*Com*_*peri-urban*_” = people living in the peri-urban commune Duyen Ha 5 km away from the city along the Red River; (ii) “*Com*_*urban*_” = people living in the urban area of Hanoi, in Bang B village or Tam Hiep commune along the To Lich River and potential exposed to wastewater; (iii) “*Farmer*_*peri-urban*_” = peri-urban farmers living in Duyen Ha commune using the irrigation water from Red River, wells or local drains, which are not contaminated with the city’s wastewater; (iv) “*Farmer*_*urban*_” = urban farmers living in Bang B village or Tam Hiep commune reusing wastewater from To Lich River; and (v) “*Worker*_*HSDC*_” = workers from Hanoi Sewerage and Drainage Company (HSDC) maintaining drainage channels and operating the Yen So treatment plants

The prevalence of infection with any intestinal parasite among *Farmer*_*peri-urban*_, *Farmer*_*urban*_, *Com*_*urban*_, *Worker*_*HSDC*_ and *Com*_*peri-urban*_ was 30 %, 11 %, 10 %, 10 % and 7 %, respectively (Table 4 and Fig. 3). Only 1 % of the participants was found with multiple intestinal parasitic infections. The highest prevalence of soil-transmitted helminths was found in *Farmer*_*peri-urban*_ (25 % for hookworm and 5 % for *T. trichiura*). *Ascaris lumbricoides* was only detected in *Com*_*urban*_ and *Worker*_*HSDC*_; a prevalence of 2 % and 1 %, respectively. Infections with soil-transmitted helminths were of light intensity [37]. The prevalence of intestinal protozoa was low; only nine infections with *B. coli*, *E. coli* and *G. intestinalis* were found, resulting to an overall prevalence of 1.2 %.

**Table 4:** Prevalence and intensity of parasite infections among the participants enrolled in the cross-sectional survey in Hanoi, stratified by five exposure groups in the Than Tri district, Hanoi, between April and June 2014

Prevalence of infection/Exposure groups^a^	*Com*_*peri-urban*_		*Com*_*urban*_		*Farmer*_*peri-urban*_		*Farmer*_*urban*_		*Worker*_*HSDC*_		Chi-square test
	*N* = 101		*N* = 170		*N* = 129		*N* = 153		*N* = 128		
	*n*	%^d^	*n*	%^c^	*n*	%^c^	*n*	%^c^	*n*	%^c^	*P*-value
Intestinal parasite^b^	7	6.9	17	10.0	39	30.2	17	11.1	13	10.2	< 0.001
Soil-transmitted helminth^c^	6	5.9	16	9.4	39	30.2	15	9.8	11	8.6	< 0.001
Intestinal protozoa	1	1.0	1	0.6	2	1.6	2	1.3	2	1.6	0.932
Hookworm	4	4.0	6	3.5	32	24.8	11	7.2	5	3.9	< 0.001
Light infection (1–1,999 epg)	4	4.0	6	3.5	32	24.8	11	7.2	4	3.1	< 0.001
Moderate infection (2,000–3,999 epg)	0	0.0	0	0.0	0	0.0	0	0.0	1	0.8	
*Trichuris trichiura*	2	2.0	9	5.3	7	5.4	4	2.6	9	7.0	0.281
Light infection (1–999 EPG)	2	2.0	9	5.3	7	5.4	4	2.6	8	6.3	0.384
Moderate infection (1,000–9,999 epg)	0	0.0	0	0.0	0	0	0	0	1	0.8	
*Ascaris lumbricoides*	0	0.0	2	1.2	0	0	0	0	2	1.6	0.252
Light infection (1–4,999 epg)	0	0.0	2	1.2	0	0	0	0	1	0.8	< 0.001
Moderate infection (5,000–49,999 epg)	0	0.0	0	0.0	0	0	0	0	0	0.0	< 0.001
*Giardia intestinalis*	0	0.0	1	0.6	0	0	0	0	1	0.8	0.612
*Entamoeba coli*	0	0.0	1	0.6	1	0.8	2	1.3	1	0.8	0.833
*Entamoeba histolytica/E. dispar*	0	0.0	0	0.0	0	0.0	0	0.0	0	0.0	na
*Balantidium coli*	1	1.0	0	0.0	1	0.8	0	0.0	0	0.0	0.403

^a^Exposure groups: *Com*_*peri-urban*_: people living in the peri-urban commune Duyen Ha, 5 km away from the city along the Red River; *Com*_*urban*_: people living in the urban area of Hanoi, in Bang B village or Tam Hiep commune along the To Lich River and potential exposed to wastewater; *Farmer*_*peri-urban*_: peri-urban farmers living in Duyen Ha commune using the irrigation water from Red River, wells or local drains, which are not contaminated with the city’s wastewater; *Farmer*_*urban*_: urban farmers living in Bang B village or Tam Hiep commune reusing wastewater from To Lich River; and *Worker*_*HSDC*_: workers from Hanoi Sewerage and Drainage Company (HSDC) maintaining drainage channels and operating the Yen So treatment plants

^b^Intestinal parasitic infection includes: *Ascaris lumbricoides*, *Trichuris trichiura*, hookworm and any intestinal protozoa

^c^Soil-transmitted helminth infection includes: *Ascaris lumbricoides*, *Trichuris trichiura*, hookworm

^d^Prevalence rate is calculated out of the results of the examination of a single stool sample by means of duplicate Kato-Katz and the formalin-ether concentration method, infection intensity by the examination via duplicate Kato-Katz

*Abbreviation*: *epg*, eggs per gram; *na*, not applicable

The prevalence of self-reported 14-day diarrhoea was not significantly different between study groups and ranged between 12 % (*Com*_*peri-urban*_) and 4 % (*Farmer*_*urban*_) (Table 5, Fig. 3). However, self-reported rates of skin and eye problems were significantly different between the five exposure groups. General skin problems ranged between 27 % (*Farmer*_*peri-urban*_) and 12 % (*Farmer*_*urban*_). Eye problems were most frequently reported in *Farmer*_*urban*_ (38 %), followed by *Worker*_*HSDC*_ (34 %) and *Com*_*urban*_ (29 %), whereas considerably lower rates of 22 % and 21 % were found in *Farmer*_*peri-urban*_ and *Com*_*peri-urban*_.

**Table 5:** Self-reported health outcomes experienced in the last two weeks before the interview among the participants enrolled in a cross-sectional survey stratified by five exposure groups in the Than Tri district, Hanoi, between April and June 2014

Self-reported health issues over the past 2 weeks/ Exposure group^a^	*Com*_*peri-urban*_		*Com*_*urban*_		*Farmer*_*peri-urban*_		*Farmer*_*urban*_		*Worker*_*HSDC*_		Chi-square test
	*N* = 101		*N* = 170		*N* = 129		*N* = 153		*N* = 128		
	*n*	%	*n*	%	*n*	%	*n*	%	*n*	%	*P*-value
Diarrhoea											
14-day prevalence	12	11.9	16	9.4	5	3.9	16	10.5	10	7.8	0.205
7-day prevalence	10	9.9	11	6.5	4	3.1	12	7.8	7	5.5	0.279
Number of episodes (14 days)											
1	9	8.9	12	7.1	5	3.9	12	7.8	6	4.7	0.411
2	0	0.0	3	1.8	0	0.0	1	0.7	3	2.3	
3	2	2.0	0	0.0	0	0.0	2	1.3	1	0.8	
4	1	1.0	1	0.6	0	0.0	0	0.0	0	0.0	
Eye problems (one or more symptoms)	21	20.8	50	29.4	28	21.7	58	37.9	43	33.6	0.008
Eye irritation	8	7.9	6	3.5	10	7.7	23	15.0	32	25.0	< 0.001
Sensitivity to light	2	2.0	1	0.6	0	0.0	5	3.3	3	2.3	0.172
Other eye problems	11	10.9	45	26.4	18	14.0	41	26.8	13	10.2	0.352
Skin problems (one or more symptoms)	23	22.8	32	18.8	35	27.1	18	11.8	29	22.7	0.024
Skin irritation	3	3.0	5	2.9	6	4.7	2	1.3	13	10.2	0.004
Itching	21	20.8	22	12.9	29	22.5	10	6.5	18	14.1	0.001
Other skin problems	0	0.0	10	5.9	3	2.3	10	6.5	5	4.7	0.402
Other self-reported signs and symptoms											
Headache	38	37.6	69	40.6	68	52.7	84	54.9	50	39.1	0.006
Fever	7	6.9	8	4.7	9	7.0	10	6.5	4	3.1	0.591
Abdominal pain	27	26.7	40	23.5	39	30.2	42	27.5	26	20.3	0.398
Acute coughing	25	24.8	46	27.1	39	30.2	44	28.8	40	31.3	0.822
Chronic coughing	5	5.0	15	8.8	1	0.8	14	9.2	2	1.6	0.002
Chest pain	13	12.9	30	17.6	23	17.8	30	19.6	18	14.1	0.582
Loss of weight	14	13.9	16	9.4	14	10.9	17	11.1	5	3.9	0.113
Nausea	12	11.9	16	9.4	7	5.4	15	9.8	5	3.9	0.125
Vomiting	2	2.0	3	1.8	2	1.6	3	2.0	1	0.8	0.941
Vomiting of blood	0	0.0	0	0.0	0	0.0	1	0.7	0	0.0	0.485
Muscle pain	19	18.8	43	25.3	32	24.8	52	34.0	33	25.8	0.097
Back pain	48	47.5	80	47.1	77	59.7	102	66.7	45	35.2	< 0.001
Joint pain	30	29.7	74	43.5	68	52.7	91	59.5	29	22.7	< 0.001
Injuries	3	3.0	8	4.7	5	3.9	8	5.2	5	3.9	0.922

^a^Exposure groups: *Com*_*peri-urban*_: people living in the peri-urban commune Duyen Ha, 5 km away from the city along the Red River; *Com*_*urban*_: people living in the urban area of Hanoi, in Bang B village or Tam Hiep commune along the To Lich River and potential exposed to wastewater; *Farmer*_*peri-urban*_: peri-urban farmers living in Duyen Ha commune using the irrigation water from Red River, wells or local drains, which are not contaminated with the city’s wastewater; *Farmer*_*urban*_: urban farmers living in Bang B village or Tam Hiep commune reusing wastewater from To Lich River; and *Worker*_*HSDC*_: workers from Hanoi Sewerage and Drainage Company (HSDC) maintaining drainage channels and operating the Yen So treatment plants

*Farmer*_*peri-urban*_ had the highest adjusted odds of intestinal parasitic infection compared to the other groups (aOR 5.3, 95 % CI: 2.1–13.7) (Table 6 and Fig. 3). Higher educational attainment and socioeconomic status were negatively associated with parasitic infections, though without statistical significance. Lack of access to toilet at home and not being dewormed for more than 12 months showed an almost significant positive association with intestinal parasitic infection (aOR 3.1, 95 % CI: 0.9–11.0 and aOR 2.5, 95 % CI: 0.9–7.0, respectively). By means of univariate regression analysis, higher odds for intestinal parasite infections were observed by at least a factor of 1.7 for all exposure groups when compared to *Farmer*_*peri-urban*_ (Fig. 3 and Additional file 1: Tables S1-S6). For hookworm infections, increased risks were observed among *Farmer*_*peri-urban*_ and *Farmer*_*urban*_ (OR 8.0, 95 % CI: 2.7–23.5 and 1.9, 95 % CI: 2.7–6.1, respectively). For *T. trichiura* infection, highest risks were observed in *Worker*_*HSDC*_ (OR 3.7, 95 % CI: 0.8–17.7). Risks for eye problems were highest in participants with exposure to wastewater; *Farmer*_*urban*_, *Com*_*urban*_ and *Worker*_*HSDC*_ (OR of 2.3, 95 % CI: 1.5–1.9, respectively). No trend for a difference in risk between the exposure groups was observed for 14-day diarrhoea prevalence.

**Table 6:** Results of univariate and multivariate logistic regression analysis for total parasitic infections (*Ascaris lumbricoides*, *Trichuris trichiura*, hookworm and intestinal protozoa) in a cross-sectional survey in the Than Tri district, Hanoi, between April and June 2014

Intestinal parasitic infection^a^ (total population, *N* = 681; infections 13.6 %, *n* = 93)		Infections		Univariate logistic regression^c^				Multivariate logistic regression^c^			
		*n*	%	OR	95 % CI		*P*-value^d^	aOR	95 % CI		*P*-value^d^
Exposure group^b^	*Com*_***peri-urban***_	101	6.9	1.00			< 0.001	1.00			
	*Com*_***urban***_	170	10.0	1.49	0.60	3.73	0.392	1.61	0.61	4.22	0.333
	*Farmer*_***peri-urban***_	129	30.3	5.82	2.48	13.68	< 0.001	5.30	2.05	13.69	0.001
	*Farmer*_***urban***_	153	11.1	1.68	0.67	4.21	0.269	1.72	0.60	4.91	0.314
	*Worker*_*HSDC*_	128	10.2	1.52	0.58	3.96	0.393	2.11	0.71	6.24	0.179
Sex	Male	166	12.1	1.00							
	Female	512	14.6	0.84	0.49	1.42	0.511	0.77	0.42	1.41	0.395
Age				1.02	1.01	1.04	0.001	1.01	1.00	1.03	0.122
Educational attainment	Never went to school	15	20.0	1.00			0.035				
	Primary school	92	16.3	0.78	0.20	3.10	0.723	0.67	0.15	3.03	0.604
	Secondary school	306	17.0	0.82	0.22	3.00	0.763	0.68	0.16	2.96	0.605
	Tertiary school	205	8.8	0.39	0.10	1.49	0.167	0.33	0.07	1.62	0.173
	Higher education	63	7.9	0.34	0.07	1.64	0.181	0.51	0.09	3.01	0.459
Socioeconomic status	Most poor	155	18.6	1.00			0.114				
	Poor	178	11.8	0.61	0.33	1.12	0.110	0.89	0.44	1.82	0.754
	Less poor	173	15.6	0.84	0.47	1.50	0.552	1.80	0.86	3.74	0.116
	Least poor	175	9.7	0.49	0.26	0.93	0.030	1.07	0.48	2.39	0.868
Number of people per household				0.90	0.79	1.01	0.067	0.93	0.82	1.05	0.250
Toilet facility at home	Yes	661	12.9	1.00							
	No	20	40.0	5.14	2.10	12.57	< 0.001	3.12	0.88	11.03	0.078
Toilet facility at work	Yes	458	12.1	1.00							
	No	195	17.4	1.54	0.96	2.48	0.076	0.87	0.47	1.60	0.653
Wastewater cause health issues	No	86	22.1	1.00							
	Yes	595	12.4	0.50	0.28	0.88	0.016	0.74	0.39	1.40	0.352
Flooding of living area	No	654	13.9	1.00							
	Yes	27	7.4	0.49	0.12	2.13	0.344				
Flooding of working area	No	538	13.4	1.00							
	Yes	143	14.7	1.11	0.66	1.88	0.687				
Drinking tap water	No	150	14.0	1.00							
	Yes	531	13.6	0.96	0.57	1.63	0.890				
Drinking rain water	No	628	13.4	1.00							
	Yes	53	17.0	1.32	0.62	2.81	0.464				
Drinking bore hole water	No	595	12.9	1.00							
	Yes	86	18.6	1.54	0.85	2.78	0.155	0.91	0.41	2.01	0.808
Bathing with tap water	No	90	16.7	1.00							
	Yes	591	13.2	1.69	0.71	4.00	0.232				
Bathing with rain water	No	647	13.3	1.00							
	Yes	34	20.6	1.31	0.80	2.13	0.278				
Bathing with bore hole water	No	514	12.8	1.00							
	Yes	167	16.2	1.27	0.15	10.97	0.830				
Preventive chemotherapy received in the past	< 6 months	69	7.2	1.00			*0.038*				
	6 to <12 months	77	6.5	0.89	0.25	3.21	0.857	0.83	0.20	3.42	0.798
	<12 months	493	15.6	2.37	0.92	6.08	0.073	2.53	0.92	6.95	0.072
	Never took deworming	42	14.3	2.13	0.61	7.48	0.237	1.87	0.48	7.25	0.363

^a^Intestinal parasitic infection includes: *Ascaris lumbricoides*, *Trichuris trichiura*, hookworm and any intestinal protozoa

^b^Exposure groups: *Com*_*peri-urban*_: people living in the peri-urban commune Duyen Ha, 5 km away from the city along the Red River; *Com*_*urban*_: people living in the urban area of Hanoi, in Bang B village or Tam Hiep commune along the To Lich River and potential exposed to wastewater; *Farmer*_*peri-urban*_: peri-urban farmers living in Duyen Ha commune using the irrigation water from Red River, wells or local drains, which are not contaminated with the city’s wastewater; *Farmer*_*urban*_: urban farmers living in Bang B village or Tam Hiep commune reusing wastewater from To Lich River; and *Worker*_*HSDC*_: workers from Hanoi Sewerage and Drainage Company (HSDC) maintaining drainage channels and operating the Yen So treatment plants

^c^ *P*-values were obtained from likelihood ratio tests. The core of the multivariate model included exposure group, sex, age, educational attainment, socioeconomic status and number of people per household. In addition, all risk factors with a *P*-value < 0.2 in the univariate analyses were included into the multivariate regression analysis (as indicated in the table)

^d^ *P*-values were obtained from likelihood ratio tests overall *P*-value of the respective categorical variable are indicated in italic letters

## Discussion

We report prevalence rates of, and risk factors for, intestinal parasite infections in urban and peri-urban communities that are at different levels of exposure to the wastewater reuse system in Hanoi, Vietnam. The highest prevalence of intestinal parasite infections was observed in peri-urban farmers (30 %), whereas lower prevalences (< 11 %) were found in urban farmers reusing wastewater, workers who maintain the wastewater channels and common urban and peri-urban community members. Hookworm was the predominate soil-transmitted helminth with an overall prevalence of 25 % in peri-urban farmers. Peri-urban farmers were at a significantly higher odds of intestinal parasite infection compared to other groups (aOR 5.3, 95 % CI: 2.1–13.7). The considerable risk for intestinal parasite infection in this group might be explained, at least partially, by a reported lack of access to toilet facility at home and a general lack of awareness towards the health risk in regard to wastewater among peri-urban farmers. Moreover, it was striking that 72 % of all participants reported to not having received deworming within the past 12 months before the study.

The observed differences between rural and peri-urban communities, especially in farmers, are in line with previous reports from studies in Asia and other parts of the world, indicating that urbanization is related to a decline of intestinal parasites [1, 6]. We found that at least one third of the peri-urban inhabitants rely on bore hole water as source for drinking or bathing and that 15 % of the peri-urban inhabitants had no access to toilet facilities at their home. Our findings support the conclusions of Do and colleagues who conducted a cross-sectional survey in Yen So commune in Hanoi in 2002 that revealed similar risk of intestinal parasite infections among urban farmers handling wastewater compared to peri-urban farmers [38]. However, prevalence rates of species-specific soil-transmitted helminths were considerably higher across all participants [*A. lumbricoides* (21.6 %), *T. trichiura* (9.8 %) and hookworm (21.8 %)] in [38], as compared to prevalences of 0.4 %, 4.4 % and 8.4 %, respectively, observed in our study. These considerably lower rates might suggest that the various improvements due to education and socioeconomic development in face of urbanization helped to bring down the prevalence of intestinal parasites over the last decade. Another reason is that people in many parts of Southeast Asia are being targeted by preventive chemotherapy against soil-transmitted helminthiasis and other neglected tropical diseases [39, 40]. The low prevalence of *A. lumbricoides* and *T. trichiura* infections correlates with concentrations of < 1 egg/l found in the environment and the presumed low infection risk of *A. lumbricoides* and *T. trichiura* [29]. However, the absence of hookworm eggs does not correlate with the respective prevalence in the exposure groups, especially in peri-urban farmers [29]. This may be explained by the fact that only hookworm eggs in water were assessed, while larval stages and eggs in soil or sediments were not [41]. Another reason for hookworm transmission could be open defecation, which is mainly practised by peri-urban farmers, due to a lack of access to toilet facilities at home and at work [9]. Overall, the prevalence of intestinal protozoa detected in the current study (< 2 %) was considerably lower than what has been reported from rural communities along Nhue River in Hanam province [11]. However, other intestinal protozoa species that were not detected by our diagnostic approach, such as *Cryptosporidium* spp. and *Cyclospora* spp., may be of importance [42]. The higher prevalence of diarrhoea, skin and eye diseases in farmers and workers exposed to wastewater compared to other groups is in line with reports from other studies conducted around Hanoi and along sanitation chains of urban and peri-urban settings [23, 24, 32]. Hence, further risk profiling such as quantitative microbial risk assessment (QMRA) or chemical risk assessments should be pursued for specific causative hazards (i.e. pathogenic bacteria, viruses and toxic chemicals, such as heavy metals, pesticides and fertilizers).

Our study has several limitations. First, the general attendance was lower than anticipated, and hence, we did not achieve the intended sample size. Results must be interpreted with caution. Secondly, most of the participants were females aged 40 years and above. Hence, our sample is not representative of the general population. However, it is representative for Hanoi’s farmers as farming activities in urban and peri-urban communities are indeed mostly carried out by older women [43]. Thirdly, a single stool sample was examined, and hence, the point-prevalence rates of helminth and intestinal protozoa infections were underestimated [44]. In order to increase the sensitivity and to have a more precise understanding of the diversity of pathogenic organisms, multiple stool samples and a suite of highly sensitive diagnostic approaches such as polymerase chain reaction (PCR) or a metagenomics approach should be considered [45, 46]. Fourthly, since this study only reflects one point in time, i.e. the rainy season, we may have missed seasonal outbreaks of typhoid, cholera and other diseases. More generally, there might be seasonal patterns of intestinal parasite infections, not captured by our study design [47–49]. Finally, it has been shown that self-reported disease outcomes (e.g. diarrhoea, skin and eye problems) are prone to reporting bias. Hence, longitudinal monitoring of diarrhoea incidence by well-trained health personnel are warranted to obtain a more accurate understanding [50].

Despite these limitations, our findings raise a number of important issues. First, even though the risk of parasite infection was relatively low, other pathogenic organisms such as viruses or bacteria may be transmitted directly or indirectly via the crops and fish produced with wastewater, which may give rise to diarrhoea, skin and eye diseases as reported by the participants of our study [16]. Secondly, even though we found low prevalence in adults, intestinal parasite infections may be a health issue in school-aged children in these settings, as children may play in agriculture fields or swim in ponds fed with wastewater. This is underlined by a study published in 2004, which detected a high prevalence rate in schoolchildren (77 %), particularly *T. trichiura* (67 %) and *A. lumbricoides* (34 %), in the area around Hanoi [51]. Thirdly, integrated strategies to control or eliminate intestinal parasitic infections in such urban and peri-urban transition zones are needed [52, 53]. For example, adapted risk analysis frameworks and transmission assessment surveys of intestinal parasitic infections to break transmission cycles and approach local elimination of intestinal parasitic infections [54].

## Conclusions

Taken together, our results suggest that peri-urban farmers are at higher risk of intestinal parasitic infections than their urban counterparts, even though exposure to highly contaminated wastewater is less common. Peri-urban communities, located only 5 km away from the urban area have limited access to improved sanitation and lack awareness towards health risks of exposure to contained water, which is associated with a high prevalence of intestinal parasitic infections. We recommend further quantitative risk assessments of microbial and chemical hazards and transmission assessment surveys of intestinal parasite infections, diarrhoeal, skin and eye diseases. Hence, there is a need for the implementation of control strategies to break transmission cycles, approach local elimination of parasitic infections and reduce risk for diarrhoea in urban and peri-urban transition zones in Hanoi and other cities in Southeast Asia.

## Additional file

Additional file 1: Univariate logistic regression models for intestinal parasitic infections and self-reported signs. **Table S1.** Results of univariate logistic regression analysis for soil-transmitted helminth infections (*Ascaris lumbricoides*, *Trichuris trichiura* and hookworm) in a cross-sectional survey in the Than Tri district, Hanoi, between April and June 2014. **Table S2.** Results of univariate logistic regression analysis for *Trichuris trichiura* infections in a cross-sectional survey in the Than Tri district, Hanoi, between April and June 2014. **Table S3.** Results of univariate logistic regression analysis for hookworm infections in a cross-sectional survey in the Than Tri district, Hanoi, between April and June 2014. **Table S4.** Results of univariate logistic regression analysis for self-reported 14-days diarrhoea in a cross-sectional survey in the Than Tri district, Hanoi, between April and June 2014. **Table S5.** Results of univariate logistic regression analysis for self-reported skin problems in a cross-sectional survey in the Than Tri district, Hanoi, between April and June 2014. **Table S6.** Results of univariate logistic regression analysis for self-reported eye problems in a cross-sectional survey in the Than Tri district, Hanoi, between April and June 2014. (DOCX 106 kb)
