# Supplementary material for: Intestinal parasite infections and associated risk factors in communities exposed to wastewater in urban and peri-urban transition zones in Hanoi, Vietnam
Source: Parasit Vectors. 2016 Oct 10;9:537. doi: 10.1186/s13071-016-1809-6 (PMC5057403; doi:10.1186/s13071-016-1809-6)
Supplement: Additional file 1: — Univariate logistic regression models for intestinal parasitic infections and self-reported signs. Table S1. Results of univariate logistic regression analysis for soil-transmitted helminth infections (Ascaris lumbricoides, Trichuris trichiura and hookworm) in a cross-sectional survey in the Than Tri district, Hanoi, between April and June 2014. Table S2. Results of univariate logistic regression analysis for Trichuris trichiura infections in a cross-sectional survey in the Than Tri district, Hanoi, between April and June 2014. Table S3. Results of univariate logistic regression analysis for hookworm infections in a cross-sectional survey in the Than Tri district, Hanoi, between April and June 2014. Table S4. Results of univariate logistic regression analysis for self-reported 14-days diarrhoea in a cross-sectional survey in the Than Tri district, Hanoi, between April and June 2014. Table S5. Results of univariate logistic regression analysis for self-reported skin problems in a cross-sectional survey in the Than Tri district, Hanoi, between April and June 2014. Table S6. Results of univariate logistic regression analysis for self-reported eye problems in a cross-sectional survey in the Than Tri district, Hanoi, between April and June 2014. (DOCX 106 kb) [file 13071_2016_1809_MOESM1_ESM.docx]

**Additional file 1.** Univariate logistic regression models intestinal parasitic infections and self-reported signs

**Table S1** Results of univariate logistic regression analysis for soil-transmitted helminth infections (*Ascaris lumbricoides*, *Trichuris trichiura* and hookworm) in a cross-sectional survey in the Than Tri district, Hanoi, between April and June 2014

| Soil-transmitted helminth infections^§^  (total population, *N* = 681; infections *n* = 87) | |  | Univariate logistic regression* | | | |
| --- | --- | --- | --- | --- | --- | --- |
|  |  |  | OR | 95% CI | | *P*-value |
| Exposure group** | *Com****_peri-urban_*** |  |  |  |  | 0.000 |
|  | *Com****_urban_*** |  | 1.65 | 0.62 | 4.35 | 0.316 |
|  | *Farmer****_peri-urban_*** |  | 6.86 | 2.77 | 16.99 | <0.001 |
|  | *Farmer****_urban_*** |  | 1.72 | 0.64 | 4.60 | 0.279 |
|  | *Worker_HSDC_* |  | 1.49 | 0.53 | 4.17 | 0.449 |
| Sex | Male |  |  |  |  |  |
|  | Female |  | 0.79 | 0.45 | 1.37 | 0.395 |
| Age |  |  | 1.02 | 1.01 | 1.04 | 0.004 |
| Educational attainment | Never went to school |  |  |  |  | 0.030 |
|  | Primary |  | 0.72 | 0.18 | 2.87 | 0.640 |
|  | Secondary |  | 0.74 | 0.20 | 2.74 | 0.657 |
|  | Higher education |  | 0.36 | 0.09 | 1.36 | 0.132 |
| Socio-economic status | Most poor |  |  |  |  | 0.181 |
|  | Poor |  | 0.66 | 0.35 | 1.24 | 0.195 |
|  | Less poor |  | 0.92 | 0.51 | 1.67 | 0.784 |
|  | Least poor |  | 0.52 | 0.27 | 1.02 | 0.058 |
| Number of people per household | 1 to 4 |  |  |  |  | 0.342 |
|  | 4 to 6 |  | 0.68 | 0.40 | 1.17 | 0.166 |
|  | > 6 |  | 0.67 | 0.36 | 1.27 | 0.222 |
| Toilet facility at home | Yes |  |  |  |  |  |
|  | No |  | 5.60 | 2.28 | 13.71 | <0.001 |
| Toilet facility at work | Yes |  |  |  |  | 0.198 |
|  | No |  | 1.54 | 0.96 | 2.47 | 0.102 |
| Wastewater can cause health issues | No |  |  |  |  |  |
|  | Yes |  | 0.46 | 0.26 | 0.80 | 0.073 |
| Flooding of living area | No |  |  |  |  |  |
|  | Yes |  | 0.54 | 0.12 | 2.30 | 0.401 |
| Flooding of working area | No |  |  |  |  |  |
|  | Yes |  | 0.98 | 0.56 | 1.70 | 0.940 |
| Drinking tap water | No |  |  |  |  |  |
|  | Yes |  | 0.94 | 0.55 | 1.60 | 0.817 |
| Drinking rain water | No |  |  |  |  |  |
|  | Yes |  | 1.44 | 0.68 | 3.07 | 0.342 |
| Drinking bore hole water | No |  |  |  |  |  |
|  | Yes |  | 1.53 | 0.83 | 2.82 | 0.168 |
| Bath tap water | No |  |  |  |  |  |
|  | Yes |  | 0.77 | 0.41 | 1.42 | 0.398 |
| Bath rain water | No |  |  |  |  |  |
|  | Yes |  | 1.84 | 0.77 | 4.36 | 0.167 |
| Bath bore hole water | No |  |  |  |  |  |
|  | Yes |  | 1.37 | 0.16 | 11.86 | 0.775 |
| Preventive chemotherapy received in the past | < 6 months |  |  |  |  | 0.084 |
|  | 6 to <12 months |  | 0.89 | 0.25 | 3.21 | 0.857 |
|  | <12 months |  | 2.15 | 0.84 | 5.54 | 0.111 |
|  | Never took deworming |  | 2.13 | 0.61 | 7.48 | 0.237 |

^§^soil-transmitted helminth infections include: *Ascaris lumbricoides*, *Trichuris trichiura*, hookworm. **P*-values were obtained from likelihood ratio tests. **(i)“*Com _peri-urban_*” = people living in the peri-urban commune Duyen Ha, 5 km away from the city along the Red River; (ii) “*Com _urban_*” = people living in the urban area of Hanoi, in Bang B village or Tam Hiep commune along the To Lich River and potential exposed to wastewater; (iii) “*Farmer _peri-urban_*” = peri-urban farmers living in Duyen Ha commune using the irrigation water from Red River, wells or local drains, which are not contaminated with the city’s wastewater; (iv) “*Farmer _urban_*” = urban farmers living in Bang B village or Tam Hiep commune reusing wastewater from To Lich River; and (v) “*Worker _HSDC_*” = workers from Hanoi Sewerage and Drainage Company (HSDC) maintaining drainage channels and operating the Yen So treatment plants.

**Table S2** Results of univariate logistic regression analysis for *Trichuris trichiura* infections in a cross-sectional survey in the Than Tri district, Hanoi, between April and June 2014

| *Trichuris trichiura* infection  (total population, *N* = 681; infections *n* = 31) | |  | Univariate logistic regression* | | | |
| --- | --- | --- | --- | --- | --- | --- |
|  |  |  | OR | 95% CI | | *P*-value |
| Exposure group** | *Com****_peri-urban_*** |  |  |  |  | 0.242 |
|  | *Com****_urban_*** |  | 2.77 | 0.59 | 13.07 | 0.199 |
|  | *Farmer****_peri-urban_*** |  | 2.84 | 0.58 | 13.98 | 0.199 |
|  | *Farmer****_urban_*** |  | 1.33 | 0.24 | 7.39 | 0.745 |
|  | *Worker_HSDC_* |  | 3.74 | 0.79 | 17.73 | 0.096 |
| Sex | Male |  |  |  |  |  |
|  | Female |  | 1.33 | 0.60 | 2.96 | 0.486 |
| Age |  |  | 0.98 | 0.96 | 1.01 | 0.233 |
| Educational attainment | Never went to school |  |  |  |  |  |
|  | Primary |  | n.a. |  |  |  |
|  | Secondary |  | n.a. |  |  |  |
|  | Higher education |  | n.a. |  |  |  |
| Socio-economic status | Most poor |  |  |  |  | 0.052 |
|  | Poor |  | 0.87 | 0.21 | 3.53 | 0.843 |
|  | Less poor |  | 3.07 | 0.98 | 9.61 | 0.055 |
|  | Least poor |  | 2.29 | 0.70 | 7.45 | 0.169 |
| Number of people per household | 1 to 4 |  |  |  |  |  |
|  | 4 to 6 |  | 1.58 | 0.57 | 4.33 | 0.377 |
|  | > 6 |  | 1.44 | 0.46 | 4.51 | 0.527 |
| Toilet facility at home | Yes |  |  |  |  | 0.655 |
|  | No |  | 1.05 | 0.14 | 8.09 | 0.963 |
| Toilet facility at work | Yes |  |  |  |  |  |
|  | No |  | 0.72 | 0.30 | 1.69 | 0.447 |
| Wastewater can cause health issues | No |  |  |  |  |  |
|  | Yes |  | 1.37 | 0.41 | 4.59 | 0.614 |
| Flooding of living area | No |  |  |  |  |  |
|  | Yes |  | 1.00 |  |  |  |
| Flooding of working area | No |  |  |  |  |  |
|  | Yes |  | 0.90 | 0.36 | 2.23 | 0.818 |
| Drinking tap water | No |  |  |  |  |  |
|  | Yes |  | 1.49 | 0.56 | 3.96 | 0.420 |
| Drinking rain water | No |  |  |  |  |  |
|  | Yes |  | 1.29 | 0.38 | 4.38 | 0.688 |
| Drinking bore hole water | No |  |  |  |  |  |
|  | Yes |  | 0.22 | 0.03 | 1.65 | 0.141 |
| Bath tap water | No |  |  |  |  |  |
|  | Yes |  | 1.03 | 0.35 | 3.01 | 0.958 |
| Bath rain water | No |  |  |  |  |  |
|  | Yes |  | 1.33 | 0.30 | 5.83 | 0.704 |
| Bath bore hole water | No |  |  |  |  |  |
|  | Yes |  | 1.00 |  |  |  |
| Preventive chemotherapy received in the past | < 6 months |  |  |  |  | 0.836 |
|  | 6 to <12 months |  | 0.89 | 0.21 | 3.70 | 0.873 |
|  | <12 months |  | 0.76 | 0.25 | 2.27 | 0.622 |
|  | Never took deworming |  | 0.40 | 0.04 | 3.67 | 0.415 |

* *P*-values were obtained from likelihood ratio tests. **(i)“*Com _peri-urban_*” = people living in the peri-urban commune Duyen Ha, 5 km away from the city along the Red River; (ii) “*Com _urban_*” = people living in the urban area of Hanoi, in Bang B village or Tam Hiep commune along the To Lich River and potential exposed to wastewater; (iii) “*Farmer _peri-urban_*” = peri-urban farmers living in Duyen Ha commune using the irrigation water from Red River, wells or local drains, which are not contaminated with the city’s wastewater; (iv) “*Farmer _urban_*” = urban farmers living in Bang B village or Tam Hiep commune reusing wastewater from To Lich River; and (v) “*Worker _HSDC_*” = workers from Hanoi Sewerage and Drainage Company (HSDC) maintaining drainage channels and operating the Yen So treatment plants.

**Table S3** Results of univariate logistic regression analysis for hookworm infections in a cross-sectional survey in the Than Tri district, Hanoi, between April and June 2014

| hookworm infections  (total population, *N* = 681; infections *n* = 58) | |  | Univariate logistic regression* | | | |
| --- | --- | --- | --- | --- | --- | --- |
|  |  |  | OR | 95% CI | | *P*-value |
| Exposure group** | *Com****_peri-urban_*** |  |  |  |  | 0.000 |
|  | *Com****_urban_*** |  | 0.89 | 0.24 | 3.22 | 0.856 |
|  | *Farmer****_peri-urban_*** |  | 8.00 | 2.73 | 23.48 | 0.000 |
|  | *Farmer****_urban_*** |  | 1.88 | 0.58 | 6.07 | 0.292 |
|  | *Worker_HSDC_* |  | 0.99 | 0.26 | 3.77 | 0.983 |
| Sex | Male |  |  |  |  |  |
|  | Female |  | 0.63 | 0.31 | 1.27 | 0.198 |
| Age |  |  | 1.04 | 1.02 | 1.05 | 0.000 |
| Educational attainment | Never went to school |  |  |  |  | 0.023 |
|  | Primary |  | 0.54 | 0.13 | 2.23 | 0.397 |
|  | Secondary |  | 0.45 | 0.12 | 1.69 | 0.236 |
|  | Higher education |  | 0.20 | 0.05 | 0.81 | 0.024 |
| Socio-economic status | Most poor |  |  |  |  | 0.019 |
|  | Poor |  | 0.59 | 0.29 | 1.18 | 0.137 |
|  | Less poor |  | 0.61 | 0.30 | 1.22 | 0.161 |
|  | Least poor |  | 0.27 | 0.11 | 0.64 | 0.003 |
| Number of people per household | 1 to 4 |  |  |  |  | 0.089 |
|  | 4 to 6 |  | 0.50 | 0.27 | 0.94 | 0.030 |
|  | > 6 |  | 0.54 | 0.26 | 1.13 | 0.104 |
| Toilet facility at home | Yes |  |  |  |  |  |
|  | No |  | 7.51 | 2.97 | 18.97 | 0.000 |
| Toilet facility at work | Yes |  |  |  |  |  |
|  | No |  | 2.18 | 1.26 | 3.77 | 0.005 |
| Wastewater can cause health issues | No |  |  |  |  |  |
|  | Yes |  | 0.33 | 0.18 | 0.62 | 0.001 |
| Flooding of living area | No |  |  |  |  |  |
|  | Yes |  | 0.85 | 0.20 | 3.70 | 0.833 |
| Flooding of working area | No |  |  |  |  |  |
|  | Yes |  | 1.22 | 0.65 | 2.29 | 0.540 |
| Drinking tap water | No |  |  |  |  |  |
|  | Yes |  | 0.79 | 0.43 | 1.47 | 0.462 |
| Drinking rain water | No |  |  |  |  |  |
|  | Yes |  | 1.41 | 0.58 | 3.46 | 0.448 |
| Drinking bore hole water | No |  |  |  |  |  |
|  | Yes |  | 2.43 | 1.27 | 4.66 | 0.007 |
| Bath tap water | No |  |  |  |  |  |
|  | Yes |  | 0.71 | 0.34 | 1.45 | 0.346 |
| Bath rain water | No |  |  |  |  |  |
|  | Yes |  | 1.93 | 0.72 | 5.20 | 0.192 |
| Bath bore hole water | No |  |  |  |  |  |
|  | Yes |  | 2.17 | 0.25 | 18.88 | 0.483 |
| Preventive chemotherapy received in the past | < 6 months |  |  |  |  | 0.012 |
|  | 6 to <12 months |  | 2.76 | 0.28 | 27.14 | 0.385 |
|  | <12 months |  | 7.50 | 1.02 | 55.24 | 0.048 |
|  | Never took deworming |  | 9.19 | 1.03 | 81.62 | 0.047 |

* *P*-values were obtained from likelihood ratio tests. **(i)“*Com _peri-urban_*” = people living in the peri-urban commune Duyen Ha, 5 km away from the city along the Red River; (ii) “*Com _urban_*” = people living in the urban area of Hanoi, in Bang B village or Tam Hiep commune along the To Lich River and potential exposed to wastewater; (iii) “*Farmer _peri-urban_*” = peri-urban farmers living in Duyen Ha commune using the irrigation water from Red River, wells or local drains, which are not contaminated with the city’s wastewater; (iv) “*Farmer _urban_*” = urban farmers living in Bang B village or Tam Hiep commune reusing wastewater from To Lich River; and (v) “*Worker _HSDC_*” = workers from Hanoi Sewerage and Drainage Company (HSDC) maintaining drainage channels and operating the Yen So treatment plants.

**Table S4** Results of univariate logistic regression analysis for self-reported 14-days diarrhoea in a cross-sectional survey in the Than Tri district, Hanoi, between April and June 2014

| 14-days diarrhoea  (total population, *N* = 681; infections *n* = 59) | |  | Univariate logistic regression* | | | |
| --- | --- | --- | --- | --- | --- | --- |
|  |  |  | OR | 95% CI | | *P*-value |
| Exposure group** | *Com****_peri-urban_*** |  |  |  |  | 0.156 |
|  | *Com****_urban_*** |  | 0.77 | 0.35 | 1.70 | 0.519 |
|  | *Farmer****_peri-urban_*** |  | 0.30 | 0.10 | 0.88 | 0.028 |
|  | *Farmer****_urban_*** |  | 0.87 | 0.39 | 1.92 | 0.723 |
|  | *Worker_HSDC_* |  | 0.63 | 0.26 | 1.52 | 0.303 |
| Sex | Male |  |  |  |  |  |
|  | Female |  | 1.51 | 0.85 | 2.69 | 0.160 |
| Age |  |  | 1.01 | 0.99 | 1.03 | 0.307 |
| Educational attainment | Never went to school |  |  |  |  | 0.174 |
|  | Primary |  | 0.23 | 0.06 | 0.90 | 0.035 |
|  | Secondary |  | 0.22 | 0.07 | 0.76 | 0.016 |
|  | Higher education |  | 0.28 | 0.08 | 0.95 | 0.042 |
| Socio-economic status | Most poor |  |  |  |  | 0.782 |
|  | Poor |  | 0.86 | 0.39 | 1.92 | 0.713 |
|  | Less poor |  | 1.27 | 0.60 | 2.68 | 0.534 |
|  | Least poor |  | 1.02 | 0.47 | 2.23 | 0.952 |
| Number of people per household | 1 to 4 |  |  |  |  | 0.965 |
|  | 4 to 6 |  | 0.91 | 0.47 | 1.78 | 0.790 |
|  | > 6 |  | 0.95 | 0.44 | 2.04 | 0.892 |
| Toilet facility at home | Yes |  |  |  |  |  |
|  | No |  | 1.00 |  |  |  |
| Toilet facility at work | Yes |  |  |  |  |  |
|  | No |  | 0.84 | 0.45 | 1.54 | 0.569 |
| Wastewater can cause health issues | No |  |  |  |  |  |
|  | Yes |  | 2.09 | 0.74 | 5.91 | 0.166 |
| Flooding of living area | No |  |  |  |  |  |
|  | Yes |  | 0.84 | 0.19 | 3.63 | 0.813 |
| Flooding of working area | No |  |  |  |  |  |
|  | Yes |  | 1.75 | 0.97 | 3.14 | 0.063 |
| Drinking tap water | No |  |  |  |  |  |
|  | Yes |  | 1.26 | 0.64 | 2.48 | 0.513 |
| Drinking rain water | No |  |  |  |  |  |
|  | Yes |  | 1.11 | 0.42 | 2.90 | 0.836 |
| Drinking bore hole water | No |  |  |  |  |  |
|  | Yes |  | 0.77 | 0.32 | 1.84 | 0.553 |
| Bath tap water | No |  |  |  |  |  |
|  | Yes |  | 0.97 | 0.44 | 2.11 | 0.935 |
| Bath rain water | No |  |  |  |  |  |
|  | Yes |  | 1.89 | 0.70 | 5.09 | 0.206 |
| Bath bore hole water | No |  |  |  |  |  |
|  | Yes |  | 2.13 | 0.24 | 18.52 | 0.494 |
| Preventive chemotherapy received in the past | < 6 months |  |  |  |  | 0.995 |
|  | 6 to <12 months |  | 1.05 | 0.34 | 3.29 | 0.933 |
|  | <12 months |  | 0.98 | 0.40 | 2.39 | 0.961 |
|  | Never took deworming |  | 1.11 | 0.29 | 4.17 | 0.883 |

* *P*-values were obtained from likelihood ratio tests. **(i)“*Com _peri-urban_*” = people living in the peri-urban commune Duyen Ha, 5 km away from the city along the Red River; (ii) “*Com _urban_*” = people living in the urban area of Hanoi, in Bang B village or Tam Hiep commune along the To Lich River and potential exposed to wastewater; (iii) “*Farmer _peri-urban_*” = peri-urban farmers living in Duyen Ha commune using the irrigation water from Red River, wells or local drains, which are not contaminated with the city’s wastewater; (iv) “*Farmer _urban_*” = urban farmers living in Bang B village or Tam Hiep commune reusing wastewater from To Lich River; and (v) “*Worker _HSDC_*” = workers from Hanoi Sewerage and Drainage Company (HSDC) maintaining drainage channels and operating the Yen So treatment plants.

**Table S5** Results of univariate logistic regression analysis for self-reported skin problems in a cross-sectional survey in the Than Tri district, Hanoi, between April and June 2014

| Self-reported skin problems  (total population, *N* = 681; infections *n* = 137) | |  | Univariate logistic regression* | | | |
| --- | --- | --- | --- | --- | --- | --- |
|  |  |  | OR | 95% CI | | *P*-value |
| Exposure group** | *Com****_peri-urban_*** |  |  |  |  | 0.015 |
|  | *Com****_urban_*** |  | 0.79 | 0.43 | 1.44 | 0.435 |
|  | *Farmer****_peri-urban_*** |  | 1.26 | 0.69 | 2.31 | 0.450 |
|  | *Farmer****_urban_*** |  | 0.45 | 0.23 | 0.89 | 0.022 |
|  | *Worker_HSDC_* |  | 0.99 | 0.53 | 1.85 | 0.983 |
| Sex | Male |  |  |  |  |  |
|  | Female |  | 1.06 | 0.69 | 1.64 | 0.781 |
| Age |  |  | 0.98 | 0.97 | 1.00 | 0.016 |
| Educational attainment | Never went to school |  |  |  |  |  |
|  | Primary |  |  |  |  |  |
|  | Secondary |  |  |  |  |  |
|  | Higher education |  |  |  |  |  |
| Socio-economic status | Most poor |  |  |  |  | 0.386 |
|  | Poor |  | 0.65 | 0.38 | 1.11 | 0.112 |
|  | Less poor |  | 0.81 | 0.48 | 1.36 | 0.423 |
|  | Least poor |  | 0.69 | 0.41 | 1.17 | 0.168 |
| Number of people per household | 1 to 4 |  |  |  |  | 0.428 |
|  | 4 to 6 |  | 0.92 | 0.58 | 1.45 | 0.716 |
|  | > 6 |  | 0.71 | 0.41 | 1.23 | 0.223 |
| Toilet facility at home | Yes |  |  |  |  |  |
|  | No |  | 1.25 | 0.45 | 3.47 | 0.669 |
| Toilet facility at work | Yes |  |  |  |  |  |
|  | No |  | 0.71 | 0.46 | 1.10 | 0.128 |
| Wastewater can cause health issues | No |  |  |  |  |  |
|  | Yes |  | 1.34 | 0.73 | 2.46 | 0.344 |
| Flooding of living area | No |  |  |  |  |  |
|  | Yes |  | 2.88 | 1.31 | 6.36 | 0.009 |
| Flooding of working area | No |  |  |  |  |  |
|  | Yes |  | 1.25 | 0.80 | 1.95 | 0.321 |
| Bath with bore hole water | No |  |  |  |  |  |
|  | Yes |  | 2.00 | 0.36 | 11.03 | 0.426 |

* *P*-values were obtained from likelihood ratio tests. **(i)“*Com _peri-urban_*” = people living in the peri-urban commune Duyen Ha, 5 km away from the city along the Red River; (ii) “*Com _urban_*” = people living in the urban area of Hanoi, in Bang B village or Tam Hiep commune along the To Lich River and potential exposed to wastewater; (iii) “*Farmer _peri-urban_*” = peri-urban farmers living in Duyen Ha commune using the irrigation water from Red River, wells or local drains, which are not contaminated with the city’s wastewater; (iv) “*Farmer _urban_*” = urban farmers living in Bang B village or Tam Hiep commune reusing wastewater from To Lich River; and (v) “*Worker _HSDC_*” = workers from Hanoi Sewerage and Drainage Company (HSDC) maintaining drainage channels and operating the Yen So treatment plants.

**Table S6** Results of univariate logistic regression analysis for self-reported eye problems in a cross-sectional survey in the Than Tri district, Hanoi, between April and June 2014

| Self-reported eye problems  (total population, *N* = 681; infections *n* = 200) | |  | Univariate logistic regression* | | | |
| --- | --- | --- | --- | --- | --- | --- |
|  |  |  | OR | 95% CI | | *P*-value |
| Exposure group** | *Com****_peri-urban_*** |  |  |  |  | 0.008 |
|  | *Com****_urban_*** |  | 1.59 | 0.89 | 2.84 | 0.120 |
|  | *Farmer****_peri-urban_*** |  | 1.06 | 0.56 | 2.00 | 0.867 |
|  | *Farmer****_urban_*** |  | 2.33 | 1.30 | 4.16 | 0.004 |
|  | *Worker_HSDC_* |  | 1.93 | 1.05 | 3.53 | 0.033 |
| Sex | Male |  |  |  |  |  |
|  | Female |  | 0.67 | 0.45 | 1.01 | 0.056 |
| Age |  |  | 1.03 | 1.02 | 1.04 | 0.000 |
| Educational attainment | Never went to school |  |  |  |  | 0.047 |
|  | Primary |  | 0.34 | 0.11 | 1.04 | 0.058 |
|  | Secondary |  | 0.27 | 0.09 | 0.79 | 0.017 |
|  | Higher education |  | 0.24 | 0.08 | 0.70 | 0.009 |
| Socio-economic status | Most poor |  |  |  |  | 0.167 |
|  | Poor |  | 0.63 | 0.39 | 1.02 | 0.059 |
|  | Less poor |  | 0.97 | 0.61 | 1.54 | 0.908 |
|  | Least poor |  | 0.75 | 0.47 | 1.20 | 0.228 |
| Number of people per household | 1 to 4 |  |  |  |  | 0.544 |
|  | 4 to 6 |  | 0.83 | 0.55 | 1.26 | 0.380 |
|  | > 6 |  | 1.00 | 0.63 | 1.60 | 0.996 |
| Toilet facility at home | Yes |  |  |  |  |  |
|  | No |  | 0.39 | 0.11 | 1.34 | 0.136 |
| Toilet facility at work | Yes |  |  |  |  |  |
|  | No |  | 0.83 | 0.57 | 1.20 | 0.327 |
| Wastewater can cause health issues | No |  |  |  |  |  |
|  | Yes |  | 1.66 | 0.96 | 2.88 | 0.068 |
| Flooding of living area | No |  |  |  |  |  |
|  | Yes |  | 1.01 | 0.44 | 2.35 | 0.976 |
| Flooding of working area | No |  |  |  |  |  |
|  | Yes |  | 1.09 | 0.73 | 1.63 | 0.679 |
| Bath bore with hole water | No |  |  |  |  |  |
|  | Yes |  | 0.48 | 0.06 | 4.12 | 0.502 |

* *P*-values were obtained from likelihood ratio tests. **(i)“*Com _peri-urban_*” = people living in the peri-urban commune Duyen Ha, 5 km away from the city along the Red River; (ii) “*Com _urban_*” = people living in the urban area of Hanoi, in Bang B village or Tam Hiep commune along the To Lich River and potential exposed to wastewater; (iii) “*Farmer _peri-urban_*” = peri-urban farmers living in Duyen Ha commune using the irrigation water from Red River, wells or local drains, which are not contaminated with the city’s wastewater; (iv) “*Farmer _urban_*” = urban farmers living in Bang B village or Tam Hiep commune reusing wastewater from To Lich River; and (v) “*Worker _HSDC_*” = workers from Hanoi Sewerage and Drainage Company (HSDC) maintaining drainage channels and operating the Yen So treatment plants.
